# Computational Analyses of Spectral Trees from Electrospray Multi-Stage Mass Spectrometry to Aid Metabolite Identification

**DOI:** 10.3390/metabo3041036

**Published:** 2013-10-31

**Authors:** Mingshu Cao, Karl Fraser, Susanne Rasmussen

**Affiliations:** AgResearch Grasslands Research Centre, Palmerston North 4442, New Zealand; E-Mails: karl.fraser@agresearch.co.nz (K.F.); susanne.rasmussen@agresearch.co.nz (S.R.)

**Keywords:** ESI fragmentation, peak annotation, metabolite identification, *Lolium perenne*

## Abstract

Mass spectrometry coupled with chromatography has become the major technical platform in metabolomics. Aided by peak detection algorithms, the detected signals are characterized by mass-over-charge ratio (*m/z*) and retention time. Chemical identities often remain elusive for the majority of the signals. Multi-stage mass spectrometry based on electrospray ionization (ESI) allows collision-induced dissociation (CID) fragmentation of selected precursor ions. These fragment ions can assist in structural inference for metabolites of low molecular weight. Computational investigations of fragmentation spectra have increasingly received attention in metabolomics and various public databases house such data. We have developed an R package “iontree” that can capture, store and analyze MS^2^ and MS^3^ mass spectral data from high throughput metabolomics experiments. The package includes functions for ion tree construction, an algorithm (distMS2) for MS^2^ spectral comparison, and tools for building platform-independent ion tree (MS^2^/MS^3^) libraries. We have demonstrated the utilization of the package for the systematic analysis and annotation of fragmentation spectra collected in various metabolomics platforms, including direct infusion mass spectrometry, and liquid chromatography coupled with either low resolution or high resolution mass spectrometry. Assisted by the developed computational tools, we have demonstrated that spectral trees can provide informative evidence complementary to retention time and accurate mass to aid with annotating unknown peaks. These experimental spectral trees once subjected to a quality control process, can be used for querying public MS^2^ databases or *de novo* interpretation. The putatively annotated spectral trees can be readily incorporated into reference libraries for routine identification of metabolites.

## 1. Introduction

Mass spectrometry, either direct infusion-based (DIMS) or in combination with liquid chromatography (LCMS), has become the major technical platform in metabolomics due to its high sensitivity, high throughput and direct applicability to complex biological extracts. Signals detected by LCMS are characterized as peaks, the pair of mass-over-charge ratio (*m/z*) and retention time (RT), or simply the *m/z* alone when DIMS is employed. It is a challenging task to translate these signals into metabolites. Mass spectrometry-based metabolite identification has received much attention recently and common practices appear to be emerging [1,2,3,4,5]. Ultimate structural elucidation of the detected peaks with complete structural details is time consuming and impractical in metabolomics. Four levels of metabolite identification have been recommended by the Metabolomics Standards Initiative (MSI) [6], thus providing guidelines for routine metabolomics studies. Level 1 identification requires experimental spectral information to be matched with that of authentic compounds. However, because of the lack of authentic compounds in metabolomics to support the identification of all the detected chemical species [4], the MSI level 2 identification can be more practically achieved, which refers to that compounds can be putatively identified based on physiochemical properties and/or spectral similarities with public/commercial spectral libraries, without reference to authentic chemical standards. Although the chemical identities of all the detected peaks may not be required to address some biological questions, a collection of evidence that supports the annotation of detected signals should be provided [6,7]. Accurate mass measurements and other spectral features such as chromatographic behavior, isotopic ratios, and adduct ions can provide such useful structural information for the annotation of detected signals [8].

Spectral trees (or ion trees, used interchangeably hereafter) have been shown to provide unique structural information to annotate peaks. Spectral tree data are generated by multi-stage mass spectrometry (MS^n^, n > 1). Briefly, metabolites are ionized in the ion-source of the mass spectrometer by electrospray ionization (ESI, in this study), then ions can be automatically selected for isolation and fragmentation via collision with gas molecules (collision-induced dissociation, CID) to generate tandem mass spectra (denoted as MS^2^ or MS/MS). Further stages (MS^n^, n > 2) of ion fragmentation can be achieved if sufficient precursor ions can be captured. MS^n^ (n > 2) spectra when available are considered to provide greater discriminating power for more precise compound identification [9,10]. MS^2^ spectra have been routinely applied in LCMS-based proteomics for peptide sequencing, and have been the subject of extensive computational research [11]. Compared with the method development in proteomics, the bioinformatics research of metabolite fragmentation patterns imposes different challenges because small molecules often give rise to much more sparse fragmentation spectra [12] and the structural diversity of metabolites is enormous. Computational methods have recently been developed to process and quantify fragmentation data specific to small molecules [13,14]. The utilization of MS^n^ spectral fragmentation for metabolite annotation has been the subject of much recent research [1,7,15,16,17], where spectral trees were demonstrated to be highly reproducible features useful for the annotation of detected peaks. Experimental spectral trees when compared with MS^2^ spectral databases [18,19] or even *in silico* fragmentation patterns [5,10,20] can facilitate the annotation process.

Although public databases that archive fragmentation spectral data are available our experience suggests that these are not comprehensive enough to cover all metabolites detected by LCMS. Modern MS instrumentation can rapidly collect a large number of ion fragmentation spectra, which makes it a non-trivial task to mine all the spectral trees collected over various projects. Therefore, it is important to statistically analyze experimental spectral data particularly given their noisy nature, and to compare the fragmentation data generated over different projects in order to probe into the chemical identity of measured ions. With an ultimate aim to understanding the metabolome of ryegrass (*Lolium perenne*) we initially developed computational methods to analyze experimental fragmentation data (MS^2^/MS^3^) collected from DIMS based on low resolution ion trap mass spectrometry to classify and annotate nominal mass ions [21,22]. We developed computational methods using R [23] and built a package (“iontree”) to facilitate the computational investigation of all ion trees collected from various research projects. The package was submitted to Bioconductor in 2011, and the current version can be found at the website [24].

In this paper, we describe the basic functionalities implemented in the “iontree” package that was primarily designed for the analysis of low resolution spectral trees based on direct infusion MS (DIMS). The package includes functions for ion tree construction, algorithms for sparse MS^2^ spectral comparison, and tools for building platform-independent ion tree libraries that contain MS^2^/MS^3^ spectra. We have extended the functionalities we developed for the analysis of DIMS^n^ data to the analysis of fragmentation data (MS^2^) collected from liquid chromatography coupled with either low resolution (LC-LRMS) or high resolution MS (LC-HRMS). We demonstrate that the developed computation tools can be utilized for the systematic analysis of fragmentation data collected in various metabolomics platforms to aid peak annotation and metabolite identification.

## 2. Results and Discussion

### 2.1. Functionalities Implemented in the Iontree Package

Functions encapsulated in the package were originally designed to process DIMS^n^ data. Raw data, collected in either centroid or profile mode, were converted into platform-independent data formats such as mzXML [25]. For processing MS^n^ fragmentation spectra, the series of precursor ions (MS^n−1^) must be maintained. The precursor ion series was parsed from the data attribute of “filterline” in mzXML data converted from the raw data (Thermo Xcalibur), and maintained in a precursor matrix. For example, a series of precursor ions of one MS^3^ scan (spectrum) could be 1021.5 (MS^1^)–748.4 (MS^2^). Given a specified *m/z* (m) tolerance (δ) that depends on the resolution of the mass spectrometer, we provide a function to group all ions that are within the specified range, *i.e*., (m ± δ). For example, all the available MS^3^ spectra derived from the same ancestry of precursor ions (within the tolerance) are used to compute a representative MS^3^ spectrum, where the fragment ions (±δ) are merged by taking the median of the masses (in *m/z*) and computing either the maximum or 95% quantile of the ion intensities within a mass bin. Our approach relies on the similarity of the precursor series to collect all the available scans for the analysis, which enables the evaluation of statistical variation of fragment spectra. This is different from other methods where only one fragment spectrum is singled out for structural inference [26], or where the series of precursor ions may not be kept in the pre-processing steps to retrieve MS^n^ spectra [14] making it difficult to construct an ion tree. It should be noted that our experiences were largely gained from working on ion trap CID data, Thermo LTQ (Thermo, San Jose, CA, USA). Fragmentation methods continue to evolve (e.g., high collision dissociation, electron transfer dissociation *etc.*), thus the types of fragmentation data may be different and require different methods to parse [27]; Different manufacturers may have different specifications for collecting data. However, as long as the series of precursor ions of MS^n^ scans is maintained in mzXML files (*i.e*., scan header) it is possible to parse spectral trees by following the specifications. An example of automated ion tree construction from DIMS can be found in the vignette of the “iontree” package.

Distance metrics play key roles in searching spectral databases to find clues to the identity of unknown compounds. Shared peak count is the most common matching approach [13]; it simply calculates the number of masses in common between two spectra. Others, such as dot product and correlation-based distances, compare the overall similarity of two spectra. We proposed a method to compare sparse MS^2^ spectra [21] and implemented the procedures (distMS2) in the “iontree” package with a few modifications, in which additional normalization approaches were provided. Our method requires the number of most intense ions to be specified, and the peak intensities of this selected list of ions are then normalized against either the most intense ion (base peak) or the sum of all the selected ions. The distance score between two MS^2^ spectra is the sum of the Manhattan distance between the matched ions and the normalized intensities of all those unmatched ions. It can be shown that this empirical distance measurement between two spectra, S_1_ and S_2_, satisfies some of the properties, such as non-negativity d (S_1_, S_2_) ≥ 0, symmetry d (S_1_, S_2_) = d (S_2_, S_1_) and identification mark d (S_1_, S_1_) = 0), that define a metric except for the triangle inequality. So, this is a type of quasi-metric [28].

It has been shown that scan-to-scan signal intensities of fragmentation spectra exhibit significant variations [29], but the magnitude of noise of fragmentation spectra is seldom reported nor well understood. The construction of ion trees from raw scans allows the detailed study of the variation of fragmentation spectra, for example, to evaluate the variation of MS^2^ spectra derived from the same precursor ion among biological samples to ascertain the same chemical entity is measured [22], and to evaluate the variation of MS2 spectra at the scan level (see Section 2.3) to ensure quality spectra to be identified for structural interpretation. With the design goal to mine all the ion trees collected in a metabolomics project and to deposit them into a database, we have imposed two simple quality control (QC) criteria to remove noisy spectra: (1) weak spectra tend to have large variation, we therefore remove the MS^2^ spectra with a base peak (ion) intensity <100 (arbitrary units); (2) the number of MS^3^ spectra collected from the same series of precursor ions varies among samples, currently only the ion tree with the highest number of MS^3^ spectra is identified and deposited into the database. The most branched ion tree likely captures the most information on the fragmentation pathways of a nominal mass ion. However, the proper setting of the QC criteria depends on data variation. Our “iontree” package provides tools for the initial assessment of ion trees built from all samples [22].

A relational database (“mzDB”) was designed and implemented in SQLite [30], and embedded in the “iontree” package. Two tables (“experiment” and “mz”) were designed to capture all the necessary information to make valid comparisons of ion trees and to facilitate annotation. Meta-data modeled by the table “experiment” currently include information on biological origin such as species, tissues and experimental treatments applied; along with information on analytical methods such as extraction methods, chromatography conditions, ionization methods, polarity, collision energy and data pre-processing methods. Table “mz” was designed to record *m/z*, RT, MS^2^/MS^3^ spectra and custom annotation. The database schema can be found in the file “mzDBSchema.sql” within the package. The schema can be easily extended to accommodate other information that may be of particular interest, such as MS^n^ (for n > 3) spectral data, which are usually collected from targeted analyses of the selected ions.

The lack of comparative studies and standardized ESI-based fragment spectral libraries limits robust compound identification based on fragmentation data. Open-source computational tools and resources developed in our study enables comparative analysis of ion trees generated from different platforms or for different studies; and allows better use of fragmentation patterns for reliable metabolite annotation and identification.

### 2.2. Direct infusion Low Resolution Ion Trap Mass Spectrometry

Direct infusion mass spectrometry (DIMS) allows fast screening and fingerprinting of complex biological samples [31,32]. In a DIMS study with a LRMS instrument, where only nominal *m/z* features can be obtained, the MS^2^/MS^3^ spectral data provide indispensable information to annotate ions as a particular class of metabolites (MSI level 3 identification [6]).

**Figure 1:**
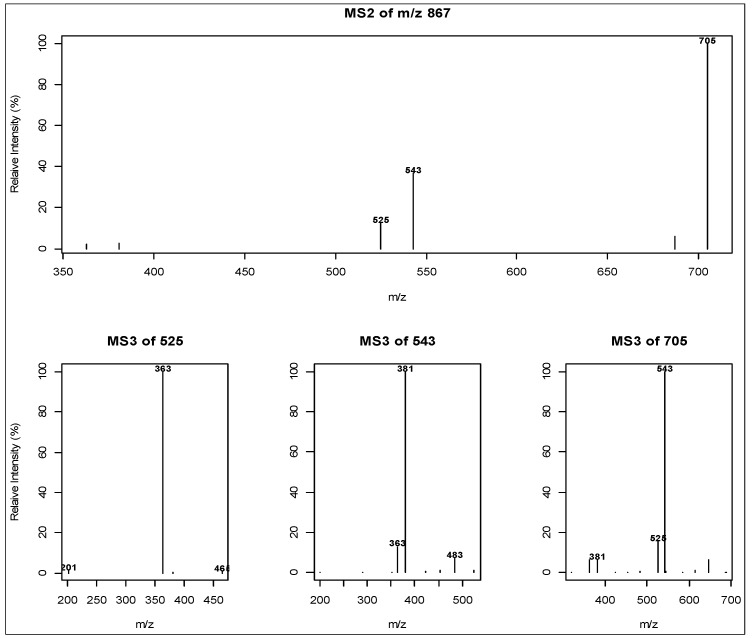
An ion tree derived from the precursor MS^1^ ion *m/z* 867 ± 0.5. The MS^2^ spectrum is the average of all MS^2^ product ion scans derived from *m/z* 867 ± 0.5, and the three MS^3^ spectra were derived from all the MS^3^ scans with the precursor series (MS^1^–MS^2^) of *m/z* 867–525, 867–543, 867–705, respectively. *m/z* 867 is an oligosaccharide with molecular mass of 828 plus a potassium adduct (penta-hexoside, [M+K]^+^). Its MS^2^/MS^3^ spectra show a series of losses of *m/z* 162 (hexose-H_2_O) supporting the proposed annotation.

To exemplify the systematic analysis of ion trees, we used the MS^2^ spectra collected from the previously published DIMS data (in positive ion mode, ESI) [21]. Of all 851 MS^1^ nominal *m/z* bins, 52% of the MS^1^ bins had MS^2^ data captured, and more than 1/3 of the MS^1^ bins rendered MS^2^ and subsequent MS^3^ data. For example, MS^2^ scans derived from the MS^1^ bin of *m/z* 867 (±0.5) were acquired for all analytical samples but not all of those gave MS^3^ scans, mainly due to insufficient ion intensity. Figure 1 shows the most branched tree of *m/z* 867 with 3 MS^3^ spectra constructed automatically from samples using the “iontree” package. Discussion of the *m/z* 867 bin on its ion type [M+K]^+^ and its relationship with other ions can be found in our previous publication [21]. MS^3^ spectra, whenever available, provide structural information on MS^2^ ions, thus more discriminating power for the identification, and should therefore be deposited in the spectral tree database.

MS^2^/MS^3^ ion trees from 447 MS^1^ nominal ions were retained after the quality control procedures (see Section 2.1) were applied. All these ion trees and the associated meta-information were deposited into the SQLite-based database, *i.e*., “mzDB”, which was included in the “iontreeData” package (Bioconductor). This database provides a persistent data repository for data curation and the annotation of mass ions based on spectral tree. As an example, the structural analysis of ion trees for *m/z* 867 allowed this nominal mass to be annotated as a penta-hexoside, [M+K]^+^, and therefore its annotation can be updated in the database.

In many situations, *de novo* interpretation of ESI fragment patterns of small compounds is beyond the reach of non-experts in the field of mass spectrometry [33]. These fragmentation spectral data, along with their interpretation, have often been published by plotting, and/or recorded in text format such as “248–206 (175, 149), 231”, which can be read as the precursor ion *m/z* 248 has major MS^2^ product ions *m/z* 206 and 231, and the MS^2^ ion *m/z* 206, in turn, gives rise to the MS^3^ product ions of *m/z* 175 and 149 [22]. By comparing the published information with the experimental spectral tree of *m/z* 248, the ion tree of which was also captured in this experiment (data not shown) and included in the “mzDB”, the nominal mass can be annotated as peramine, a pyrrolopyrazine alkaloid produced by an endophytic fungus residing in ryegrass. Although useful the data representation of ion trees in the literature makes re-use of the information difficult [12] and the information on relative intensity of fragment ions lost. Our tools allow us to record the invaluable structural information embedded in MS^2^/MS^3^ fragment spectra into a reference library for subsequent analyses. This practice enables rapid annotation of mass ions by automatically searching experimental spectral trees (mostly MS^2^) with public databases that house MS/MS data.

### 2.3. Liquid Chromatography Low Resolution Mass Spectrometry

Liquid chromatography mass spectrometry (LCMS) allows the chromatographic separation of metabolites and provides more sensitivity for metabolite identification compared with DIMS. Here we show that ion trees captured from LCMS^n^ experiments can be computed by the given range of *m/z* and retention time (RT), and that fragmentation patterns provide complementary information to RT for structural inference. We also demonstrate that distMS2 serves as an alternative metric (in comparison to a correlation-based metric) to study sparse MS^2^ spectra, providing quality fragmentation spectra for robust peak annotation.

As an example, we used data from a study of ryegrass polysaccharides [34] and investigated the variation of MS^2^ spectra derived from one MS^1^ bin (*m/z* 503 ± 0.5) corresponding to several isomeric forms of a trihexoside. 145 ryegrass sheath samples were analyzed in negative ionization mode and 3636 MS^2^ spectra were acquired from 126 samples across the elution time (20 min). Wavelet-based peak detection [35] was conducted to identify peaks with the extracted ion chromatogram (XIC) of *m/z* 503. Four chromatographically separated peaks with *m/z* 503 were identified among the samples. On average, the four peaks eluted at RT 300 [281,313], 336 [314,345], 378 [345,391] and 402 [392,414], respectively, with the median time in seconds (s) and the time range specified in brackets. Relative standard deviation (RSD) of the RTs for the four peaks was 1.4, 1.2, 1.6, and 0.8%, respectively. The expression of the peak RT variation in terms of the time range allows the comparison of RT variation and MS^2^ spectral variation (see below). These four peaks are referred to as peak 1 (300 s), peak 2 (336 s), peak 3 (378 s) and peak 4 (402 s), hereafter.

**Figure 2:**
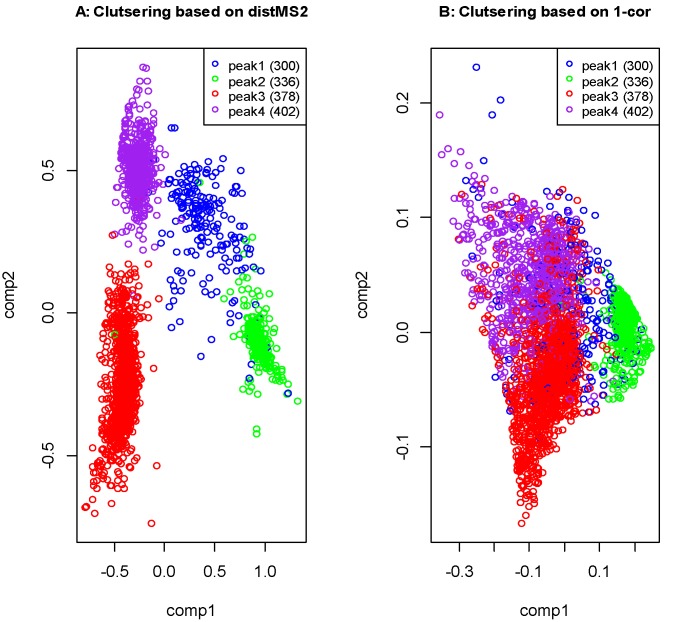
Multidimensional scaling analysis of 3039 tri-hexoside MS^2^ spectra based on distMS2 and 1-correlation coef. Retention times of all the MS^2^ scans are superimposed to show the correspondence between the fragmentation patterns and LC column behavior. (**A**) Distance metric based on distMS2. (**B**) Distance metric based on 1-r. r refers to Pearson’s correlation coefficient.

After filtering out weak MS^2^ spectra (base peak intensity <100) we compared the top five most intense ions among 3039 spectra with two distance measurements, *i.e.*, distMS2 and Pearson’s correlation coefficient (r) -based distance metric (1-r). The clustering of all the MS^2^ spectra was carried out by multidimensional scaling analysis with the results shown in Figure 2. By superimposing the retention times of the four peaks in the clustering plot it clearly indicates that the four peaks are better differentiated by distMS2 compared with 1-r. The distMS2 metric performed better probably because it takes both the ion identity and relative intensity into account while the correlation-based metric only measures the overall similarity of relative ion intensities. Additional comparisons in the context of database queries can be found in the vignette of the “iontree” package. These results suggest that distMS2 could provide an alternative way for the comparison of MS^2^ spectra, which would enable more reliable metabolite identification.

**Figure 3:**
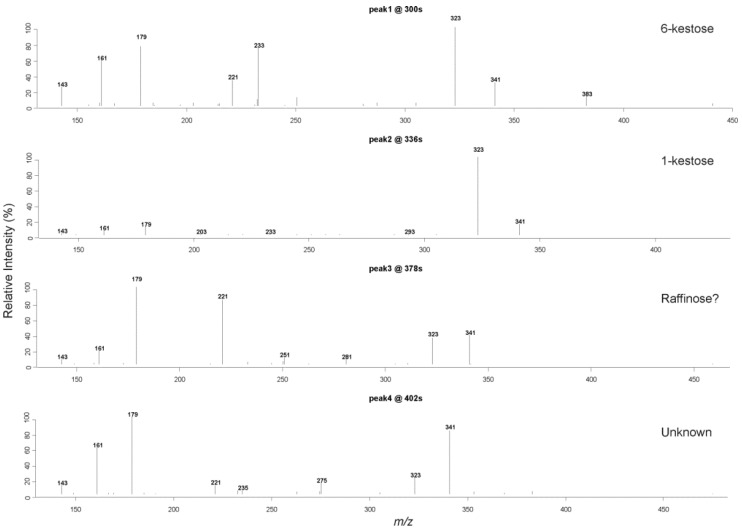
Four MS^2^ spectra retrieved from the four clusters with the strongest spectrum from each cluster plotted. The retrieved spectra provide information to infer the four isomeric peaks of *m/z* 503. With the MS^2^ spectra available from authentic compounds, peak 1 can be annotated as 6-kestose, and peak 2 as 1-kestose. Peak 3 can be putatively annotated as raffinose.

The four clusters revealed by the clustering analysis based on distMS2 (Figure 2) also suggest that *m/z* 503 corresponds to four isomers displaying the characteristic MS^2^ spectra. The most intense MS^2^ spectrum was retrieved from each of the four clusters, and plotted in Figure 3. The first two spectra were later confirmed by comparison with authentic compounds as 6-kestose (peak 1) and1-kestose (peak 2). The corresponding MS^2^ spectra of the two standards (published in Harrison *et al.* [36]) are identical with the two fragmentation patterns of peak 1 and peak 2 indicating the two peaks were positively identified (MSI Level 1). To annotate peak 3 and peak 4 we searched public MS^2^ databases including METLIN and MassBank, the results suggested peak 3 is more similar to raffinose considering the relative intensity of fragment ion *m/z* 221. These two spectra derived from peak 3 and peak 4 exhibit different ratios of fragment ions (for example *m/z* 323/341) which may reflect structural differences compared to 1-kestose and 6-kestose. Evidence from the spectral similarity allows peak 3 to be putatively identified as raffinose (MassBank ID:MT000046), but peak 4 remains classified as an unknown trisaccharide. These two spectra should therefore be deposited into the database for further annotation when more evidence becomes available, while the two verified spectra can be used directly for the routine identification of 1-kestose and 6-kestose.

This analysis not only demonstrates the power of the distMS2 metric in the context of comparing sparse MS^2^ spectra, but also provides candidate MS^2^ spectra for structural elucidation. Given the wide range of variation of MS^2^ spectra it is a non-trivial task to retrieve quality fragment spectra in high throughput settings where a large number of MS^2^ scans can be captured for a large number of samples. Unlike the ion tree construction based on DIMS^n^ data it is still difficult to automatically construct ion trees from LCMS^n^ data because the retention time must be carefully taken into consideration. With the integration of retention time information our clustering analysis based on distMS2 was able to identify quality spectra for *de novo* structural interpretation and database searching.

### 2.4. Liquid Chromatography High Resolution Mass Spectrometry

High-resolution MS^2^ data were collected from a quadrupole Orbitrap instrument, Q-Exactive (Thermo, Waltham, MA, USA) in order to investigate lipid profiles of perennial ryegrass. Ryegrass samples of a single cultivar (Grasslands Impact) were used here to illustrate the usefulness of systematic MS^2^ analysis for the annotation of lipid species.

**Figure 4:**
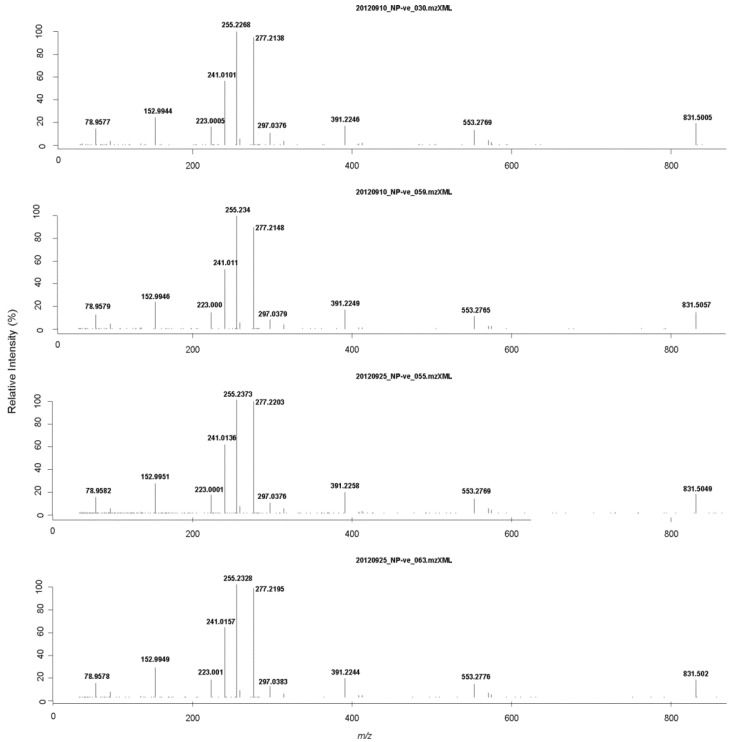
The centroid MS^2^ spectra of *m/z* 831.5050 [M-H]^−^ computed from four ryegrass samples, with the number of MS^2^ scans included in the computation from each sample being of n = 15, 14, 10, 10. The four computed MS^2^ spectra are highly correlated (with r > 0.9) and the fragment ions suggest they are highly consistent across samples (with distMS2 score < 0.15). Each of the ions labeled can be matched with the theoretical fragment ions from the Lipid Maps database. Besides the top 5 ions discussed in the text regarding the second sample, *m/z* 831.5057 is shown as the pseudo molecular ion (deprotonated), and *m/z* 78.9579 as a PO_3_ ion; *m/z* 223.000 as inositol phosphate ion with the loss of H_2_O; *m/z* 297.0379 as glycerophosphoinositol with the loss of 2H_2_O; and *m/z* 553.2765 as neutral loss of sn2 RCOOH (C18:3) group from [M-H]^−^.

From eight ryegrass samples analyzed in different analytical batches, a total of 5426 MS^2^ spectra were collected in profile mode with negative ESI setting. Profile mode data must be centroided so that fragment ions can be assigned identities (by either formula prediction or database searching). Data processing was similar to that described for the DIMS data (see Section 2.1) except that *m/z* bin width (δ) was set to 20 ppm (parts per million) for the HRMS data. Briefly, all MS^2^ spectra derived from the precursor ions within a ±20 ppm window were grouped to compute a representative MS^2^ spectrum. All fragment ions also within a ±20 ppm window were aggregated by taking the median of masses (in *m/z*) and the maximum ion intensity within the window to represent a centroid fragment spectrum. As a result, a centroid MS^2^ spectrum was computed from each sample. Plotted in Figure 4 are examples, where the centroid MS^2^ was computed from the precursor *m/z* 831.5050 (ranging from 831.5005 to 831.5057 among the four samples).

Such centroid spectra enable the robust annotation of each fragment ion. We used the centroid MS^2^ spectrum computed from the second sample (Figure 4) to search various databases including METLIN, MassBank, HMDB and LipidMaps, positive hits were obtained from the LipidMaps database only. Each of the top five most intense fragment ions matched accurately with the theoretical prediction (within 8 ppm), *i.e.*, *m/z* 255.2340 (sn1 RCOO^−^), 277.2148 (sn2 RCOO^−^), 241.0110 (inositol phosphate ion), 152.9946 (glycerol-3-phosphate ion with loss of H_2_O), and 391.2249 (neutral loss of sn2 RCOOH group, and inositol from the deprotonated ion) suggesting that the detected lipid species with *m/z* of 831.5050 can be annotated as phosphatidylinositol PI (16:0, 18:3). Other possible isomers of lipid species with the same mass, such as PI (17:2, 17:1) can be excluded based on the MS^2^ fragmentation patterns. The same procedure can be applied to mine all the 5426 MS^2^ spectra collected to classify lipid species by searching LipidMaps. This database searching is based on comparison of experimental spectra with predicted fragment ions. The relative intensity of fragment ions has not yet been taken into account for the annotation but should provide important information on the position of acyl chains and the position of double bonds in the chain. Further analysis of multi-stage spectral trees is needed to achieve more detailed structural annotation.

As shown in Figure 4, the MS^2^ spectra are consistent among the four samples. However, the examination of the XIC of *m/z* 831.5050 [M-H]^−^ among the four samples (data not shown) revealed a noticeable shift in RT (about 1 min) between analytical batches (date shown in the name of data file) due to the change of the LC column. Such RT shifts impose additional challenges for correct peak alignment and identification. Our results indicate that the MS^2^ fragment patterns might be more stable structural features than RT for annotating peaks. This important observation deserves further investigation, as if the fragmentation patterns derived from the same peak *m/z* (within the tolerance) can be determined to be algorithmically similar, this peak can be added to a set of landmark peaks [37], which can be used to guide or evaluate the peak alignment process.

## 3. Experimental Section

### 3.1. Plant Material and Analytical Methods

All data discussed in this paper were collected from various metabolomics experiments on perennial ryegrass (*Lolium perenne*). The experimental details on direct infusion mass spectrometry (DIMS) have been reported previously [21,29]; In brief, MS analysis was performed using an LTQ linear ion trap mass spectrometer (Thermo, San Jose, CA, USA) with electrospray ionization (ESI) in positive ion mode. MS^2^/MS^3^ data were collected by the isolation (2 *m/z*) and fragmentation (35% CE; relative collision energy) of the most intense ions from the MS^1^/MS^2^ spectra. Liquid chromatography coupled with low-resolution mass spectrometry was applied to the analysis of oligosaccharides in ryegrass [32]. Ryegrass sheath tissues were extracted with boiling water, and the extracts were subjected to chromatographic separation via a Thermo Hypercarb column. MS analysis was conducted using the same mass spectrometers as for DIMS but in negative ion mode. Only MS^2^ data were collected for oligosaccharide analysis. The methods developed for lipid analysis in ryegrass are unpublished yet. Very briefly, the freeze dried ryegrass blade tissues were extracted using chloroform/methanol buffer, and subjected to Thermo C1 column for the analysis of non-polar components. MS data were collected in profile mode (with negative ESI on a Q-Exactive MS (Thermo, Waltham, MA, USA) over the mass range of *m/z* 200–2,000. The mass resolution was set at 35,000 (at *m/z* 400) for both MS^1^ and MS^2^. MS^2^ data were collected with an isolation of 1.5 *m/z* and fragmentation at 30% CE from the MS^1^ spectra.

### 3.2. Software Tools and Database Resources

Details for data manipulation and analysis have been discussed in the main text. Software tools and database resources are listed here for convenient reference. The raw data (Thermo Xcalibur) collected in either centroid or profile mode were converted into mzXML by msconvert [38]. A Java library JRAP [39] or ProteoWizard toolkits [40] was used to parse mzXML data. MS^2^/MS^3^ raw data (low resolution) retrieval and analysis can be referred to in the previous publication [21]. All tools used for the analysis presented here are available from the “iontree” package [24] except for the scripts developed for the analysis of high resolution fragmentation spectra. The computational tools for high resolution MS^2^ analysis and annotated spectral tree data discussed in this paper can be made available upon request. Data inspection and statistical investigations were conducted in R [23]. The databases used for MS^2^ spectral queries were MassBank [41], METLIN [42], Lipid Maps [43] and HMDB [44].

## 4. Conclusions

In conclusion, we have developed the computational tool “iontree” as an R package to facilitate the construction of low and high resolution fragmentation data, the statistical investigation of fragmentation data and the development of in-house databases for peak annotation. As demonstrated we provided an alternative algorithm (distMS2) for the analysis of sparse MS^2^ data and pattern discovery of MS^2^ spectra, which allows subtle information to be collected and utilized for elucidating the structure of isomers. Although public databases that house MS^2^ spectral data are available, our experience suggests that these are not comprehensive enough for routine annotation by querying the large number of spectral trees generated in metabolomics studies. Our approach encourages the systematic analysis of experimental spectral trees and the compilation of quality fragment spectra (MS^2^/MS^3^) into an in-house database via a quality control process. Quality spectral trees can be interpreted manually, or used for searching against public MS^2^ data depositories or even *in silico* fragmentation data to aid metabolite identification. Spectral trees, together with retention time, accurate mass and other mass features, can be readily incorporated into reference libraries for possible routine metabolite identification.

## References

[1] Kind T., Fiehn O. (2010). Advances in structure elucidation of small molecules using mass spectrometry. Bioanal. Rev..

[2] Dunn W., Erban A., Weber R., Creek D., Brown M., Breitling R., Hankemeier T., Goodacre R., Neumann S., Kopka J. (2013). Mass appeal: Metabolite identification in mass spectrometry-focused untargeted metabolomics. Metabolomics.

[3] Draper J., Enot D., Parker D., Beckmann M., Snowdon S., Lin W., Zubair H. (2009). Metabolite signal identification in accurate mass metabolomics data with MZedDB, an interactive m/z annotation tool utilising predicted ionisation behaviour ‘rules’. BMC Bioinformatics.

[4] Wishart D.S. (2011). Advances in metabolite identification. Bioanalysis.

[5] Scheubert K., Hufsky F., Bocker S. (2013). Computational mass spectrometry for small molecules. J. Cheminform..

[6] Sumner L., Amberg A., Barrett D., Beale M., Beger R., Daykin C., Fan T.M., Fiehn O., Goodacre R., Griffin J. (2007). Proposed minimum reporting standards for chemical analysis. Metabolomics.

[7] Zhu Z.-J., Schultz A.W., Wang J., Johnson C.H., Yannone S.M., Patti G.J., Siuzdak G. (2013). Liquid chromatography quadrupole time-of-flight mass spectrometry characterization of metabolites guided by the METLIN database. Nat. Protoc..

[8] Kind T., Fiehn O. (2007). Seven Golden Rules for heuristic filtering of molecular formulas obtained by accurate mass spectrometry. BMC Bioinformatics.

[9] Olsen J.V., Mann M. (2004). Improved peptide identification in proteomics by two consecutive stages of mass spectrometric fragmentation. Proc. Natl. Acad. Sci. USA.

[10] Scheubert K., Hufsky F., Rasche F., Bocker S. (2011). Computing fragmentation trees from metabolite multiple mass mpectrometry data. J. Comput. Biol..

[11] Sadygov R.G., Cociorva D., Yates J.R. (2004). Large-scale database searching using tandem mass spectra: Looking up the answer in the back of the book. Nat. Methods.

[12] Würtinger P., Oberacher H. (2012). Evaluation of the performance of a tandem mass spectral library with mass spectral data extracted from literature. Drug Test. Anal..

[13] Benton H.P., Wong D.M., Trauger S.A., Siuzdak G. (2008). XCMS2: Processing tandem mass spectrometry data for metabolite identification and structural characterization. Anal. Chem..

[14] Gatto L., Lilley K.S. (2012). MSnbase-an R/Bioconductor package for isobaric tagged mass spectrometry data visualization, processing and quantitation. Bioinformatics.

[15] Van der Hooft J., Vervoort J., Bino R., de Vos R. (2012). Spectral trees as a robust annotation tool in LC-MS based metabolomics. Metabolomics.

[16] Ridder L., van der Hooft J.J.J., Verhoeven S., de Vos R.C.H., van Schaik R., Vervoort J. (2012). Substructure-based annotation of high-resolution multistage MSn spectral trees. Rapid Commun. Mass Spectrom..

[17] Rojas-Cherto M., Peironcely J.E., Kasper P.T., van der Hooft J.J.J., de Vos R.C.H., Vreeken R., Hankemeier T., Reijmers T. (2012). Metabolite identification using automated comparison of high-resolution multistage mass spectral trees. Anal. Chem..

[18] Tautenhahn R., Cho K., Uritboonthai W., Zhu Z., Patti G.J., Siuzdak G. (2012). An accelerated workflow for untargeted metabolomics using the METLIN database. Nat. Biotechnol..

[19] Horai H., Arita M., Kanaya S., Nihei Y., Ikeda T., Suwa K., Ojima Y., Tanaka K., Tanaka S., Aoshima K. (2010). MassBank: A public repository for sharing mass spectral data for life sciences. J. Mass Spectrom..

[20] Wolf S., Schmidt S., Muller-Hannemann M., Neumann S. (2010). In silico fragmentation for computer assisted identification of metabolite mass spectra. BMC Bioinformatics.

[21] Cao M., Koulman A., Johnson L.J., Lane G.A., Rasmussen S. (2008). Advanced data-mining strategies for the analysis of direct-infusion ion trap mass spectrometry data from the association of perennial ryegrass with Its endophytic fungus, Neotyphodium lolii. Plant Physiol..

[22] Koulman A., Cao M., Faville M., Lane G., Mace W., Rasmussen S. (2009). Semi-quantitative and structural metabolic phenotyping by direct infusion ion trap mass spectrometry and its application in genetical metabolomics. Rapid Commun. Mass Spectrom..

[23] R Development Core Team (2011). R: A Language and Environment for Statistical Computing.

[24] R package iontree. http://www.bioconductor.org/packages/release/bioc/html/iontree.html.

[25] Pedrioli P.G.A., Eng J.K., Hubley R., Vogelzang M., Deutsch E.W., Raught B., Pratt B., Nilsson E., Angeletti R.H., Apweiler R. (2004). A common open representation of mass spectrometry data and its application to proteomics research. Nat. Biotechnol..

[26] Hsieh E.J., Hoopmann M.R., MacLean B., MacCoss M.J. (2009). Comparison of database search strategies for high precursor mass accuracy MS/MS data. J. Proteome Res..

[27] Deutsch E.W., Shteynberg D., Lam H., Sun Z., Eng J.K., Carapito C., von Haller P.D., Tasman N., Mendoza L., Farrah T. (2010). Trans-Proteomic Pipeline supports and improves analysis of electron transfer dissociation data sets. Proteomics.

[28] Jain A.K., Murty M.N., Flynn P.J. (1999). Data clustering: A review. ACM Comput. Surv..

[29] Li Q., Xia Q., Wang T., Meila M., Hackett M. (2006). Analysis of the stochastic variation in LTQ single scan mass spectra. Rapid Commun. Mass Spectrom..

[30] SQLite. http://www.sqlite.org.

[31] Koulman A., Tapper B.A., Fraser K., Cao M., Lane G.A., Rasmussen S. (2007). High-throughput direct-infusion ion trap mass spectrometry: A new method for metabolomics. Rapid Commun. Mass Spectrom..

[32] Beckmann M., Parker D., Enot D.P., Duval E., Draper J. (2008). High-throughput, nontargeted metabolite fingerprinting using nominal mass flow injection electrospray mass spectrometry. Nat. Protoc..

[33] Weissberg A., Dagan S. (2011). Interpretation of ESI(+)-MS-MS spectra—Towards the identification of “unknowns”. Int. J. Mass Spectrom..

[34] Harrison S.J., Fraser K., Lane G.A., Villas-Boas S., Rasmussen S. (2009). A reverse-phase liquid chromatography/mass spectrometry method for the analysis of high-molecular-weight fructooligosaccharides. Anal. Biochem..

[35] Du P., Kibbe W.A., Lin S.M. (2006). Improved peak detection in mass spectrum by incorporating continuous wavelet transform-based pattern matching. Bioinformatics.

[36] Harrison S., Xue H., Lane G., Villas-Boas S., Rasmussen S. (2011). Linear ion trap MSn of enzymatically synthesized 13C-labeled fructans revealing differentiating fragmentation patterns of β (1–2) and β (1–6) fructans and providing a tool for oligosaccharide identification in complex mixtures. Anal. Chem..

[37] Lange E., Tautenhahn R., Neumann S., Gropl C. (2008). Critical assessment of alignment procedures for LC-MS proteomics and metabolomics measurements. BMC Bioinformatics.

[38] Kessner D., Chambers M., Burke R., Agus D., Mallick P. (2008). ProteoWizard: Open source software for rapid proteomics tools development. Bioinformatics.

[39] Bellew M., Coram M., Fitzgibbon M., Igra M., Randolph T., Wang P., May D., Eng J., Fang R., Lin C. (2006). A suite of algorithms for the comprehensive analysis of complex protein mixtures using high-resolution LC-MS. Bioinformatics.

[40] Chambers M.C., Maclean B., Burke R., Amodei D., Ruderman D.L., Neumann S., Gatto L., Fischer B., Pratt B., Egertson J. (2012). A cross-platform toolkit for mass spectrometry and proteomics. Nat. Biotechnol..

[41] MassBank. http://www.massbank.jp.

[42] METLIN. http://metlin.scripps.edu.

[43] LipidMaps. http://www.lipidmaps.org.

[44] HMDB. http://www.hmdb.ca.

